# CXCL14 Overexpression Attenuates Sepsis-Associated Acute Kidney Injury by Inhibiting Proinflammatory Cytokine Production

**DOI:** 10.1155/2020/2431705

**Published:** 2020-03-31

**Authors:** Jing Lv, Zhi-Lin Wu, Zheng Gan, Ping Gui, Shang-Long Yao

**Affiliations:** Department of Anesthesiology, Institute of Anesthesiology and Critical Care Medicine, Union Hospital, Tongji Medical College, Huazhong University of Science and Technology, 1277 Jiefang Avenue, Wuhan, Hubei 430022, China

## Abstract

CXCL14 is a relatively novel chemokine with a wide spectrum of biological activities. The present study was designed to investigate whether CXCL14 overexpression attenuates sepsis-associated acute kidney injury (AKI) in mice. Sepsis model has been established by cecal ligation and puncture (CLP). CLP induced AKI in mice as assessed by increased renal neutrophil gelatinase-associated lipocalin (NGAL) expression and serum creatinine levels. We found that renal CXCL14 expression in the kidney was significantly decreased at 12 hours after CLP. Correlation analysis demonstrated a negative association between renal CXCL14 expression and AKI markers including serum creatinine and renal NGAL. Moreover, CXCL14 overexpression reduced cytokine (TNF-*α*, IL-6, and IL-1*β*) production and NGAL expression in the kidney and decreased serum creatinine levels. *In vivo* and *in vitro* experiments found that CXCL14 overexpression inhibited M1 macrophage polarization but increased M2 polarization. Together, these results suggest that CXCL14 overexpression attenuates sepsis-associated AKI probably through the downregulation of macrophages-derived cytokine production. However, further studies are required to elucidate the underlying mechanism.

## 1. Introduction

Sepsis is a severe condition characterized by systemic inflammatory response syndrome and the presence of infection that has fatal consequences, including acute kidney injury (AKI) [1]. There is a very strong evidence that sepsis and septic shock are the most common causes of AKI in critically ill patients, accounting for more than 50% of AKI cases in the intensive care unit (ICU) [2]. Numerous evidences have also demonstrated that the development of AKI is tightly associated with poor outcome of critically ill patients [3–5]. However, the most appropriate treatment approach for this disease is still far from certain because of the conflicting data. The pathophysiology of sepsis-associated AKI is multifactorial and includes intrarenal hemodynamic changes, excessive inflammatory cytokine productions, endothelial dysfunction, and oxidative stress [6]. It is well established that proinflammatory cytokines, such as tumor necrosis factor-alpha (TNF-*α*), interleukin-1beta (IL-1*β*), and IL-6, contribute to the development of AKI in septic patients [7–9], and the inhibition of proinflammatory cytokines was able to attenuate sepsis-associated AKI and improves survival outcome [10, 11].

CXCL14 is a relatively new CXC chemokine constitutively expressed in the breast, kidney, and other epithelial tissues [12, 13]. Although the molecular mechanism governing CXCL14-mediated functions are unclear, several evidences indicate a role of CXCL14 in antitumor immunity and inflammatory response [14, 15]. It has been reported that CXCL14 expressions could be inhibited by proinflammatory cytokine TNF-*α* and endotoxin (lipopolysaccharide, LPS) [16, 17]. Since CXCL14 is constantly produced in epithelial tissues but is markedly decreased in the presence of inflammation, it has been proposed that CXCL14 plays a unique role in antimicrobial immunity prior to the establishment of inflammation [18]. In the past decades, a lot of research has been done to study CXCL14-mediated effects in cancers; however, few evidences regarding its effects in sepsis or septic shock have been reported. In the present study, we aim to test the hypothesis that CXCL14 protects animals against sepsis-associated AKI and improves survival outcome of septic animals. To study the function of CXCL14 in the development of sepsis-associated AKI, the transgenic (Tg) mice that overexpress CXCL14 (CXCL14-Tg mice) and a mouse model of sepsis by cecal ligation and puncture (CLP) were used in this study.

## 2. Materials and Methods

### 2.1. Cell Preparation and Culture

Mouse macrophage RAW264.7 cells were purchased from the American Type Culture Collection (ATCC, Manassas, VA) and were cultured in Dulbecco's Modified Eagle Medium (Gibco#11995-065) with 10% Fetal Bovine Serum and 1% penicillin-streptomycin. Cultures were maintained in humidified incubator with 5% CO_2_ at 37°C. Cells received lentivirus infections for 48 hours and were then treated with *lipopolysaccharides* (LPS,100 ng/ml, 8 h) or IL-4 (10 ng/ml, 12 h), in order to induce M1 and M2 polarization, respectively. LPS was purchased from Sigma (St. Louis, MO), and IL-4 was purchased from R&D Systems (Minneapolis, MN).

### 2.2. Lentivirus Infections

Lentivirus encoding CXCL14 open reading frame (LV-CXCL14) and a vector encoding GFP (LV-GFP) were purchased from Invitrogen (CA, USA). RAW264.7 cells were treated with lentivirus at a multiplicity of infection of 20 pfu/cell in a 2% FBS medium supplemented with 8 *μ*g/ml of polybrene (Sigma-Aldrich, USA). Then, stable transformants were selected using Blasticidin S HCl (5 *μ*M).

### 2.3. Animal Preparation and Study Design

CXCL14-Tg mice that overexpress CXCL14 were generated by a commercial company (Cyagen, Wuhan, China) as previously described [19]. Briefly, CXCL14 cDNA containing 3′ flag tag was amplified by PCR using primers including 5′-CACGAATTCCCAGCATGAGGCTCC-TGGCGGCCGC-3′ and 5′-GGAGAATTCTCACTTATCGTCGTCATCCTTGTAATCTTCTTCGTAGACCCTGCGCTTCTCG-3′ with C57BL/6 cDNA as template. Fragments were digested by EcoRI and inserted into the *kbpa* vector downstream of mouse phosphoglycerate kinase 1 (PGK) promoter. Then, the DNA fragments were purified and injected into the pronucleus of fertilized eggs of C57BL/6 mice. C57BL/6 mice were purchased from Hubei Provincial Center for Disease Control and Prevention (Wuhan, Hubei, China). Animals were maintained in individual cages under standard conditions. Animal experiments were performed in accordance with the Guidelines of Animal Use and Care and were approved by the Institutional Ethic Committee (AEC-2018-012).

Wild-type (WT) C57BL/6 mice and CXCL14-Tg mice received either sham operation or CLP after anesthesia. In total, twenty-four animals were assigned into following groups: (1) a WT sham group (sham, *n* = 6): WT C57BL/6 mice received sham operation; (2) a WT CLP group (CLP, *n* = 6): WT C57BL/6 mice received CLP without any treatments; (3) a CXCL14-Tg sham group (CXCL14-Tg, *n* = 6): CXCL14-Tg mice received sham operation; (4) a CXCL14-Tg CLP group (CXCL14 − Tg + CLP, *n* = 6): CXCL14-Tg mice received CLP without any other treatments; blood samples and kidneys were collected for biological and morphological analysis at 12 hours after CLP. In addition, another 10 animals in each group were enrolled for a 72-hour survival analysis.

### 2.4. Sepsis Model

Sepsis model was established as previously published method of CLP [20]. Briefly, a midline incision about 1 cm was made on the anterior abdomen after adequate anesthesia. The cecum was isolated and then ligated at 100% of its total length. The cecum was punctured twice with a sterile 20-G needle. The cecum was placed back, and the abdominal was closed. Animals in control groups received midline incision without CLP.

### 2.5. Measurements of Cytokines by ELISA

The kidneys were removed after perfusion with iced-cold PBS, and the renal cortex was rapidly dissected and minced on ice. Approximately 5 mg of renal tissues were placed in a microfuge tube, and 300 *μ*L of complete extraction buffer (100 mM Tris, pH 7.4, 150 mM NaCl, 1 mM EGTA, 1 mM EDTA, 1% Triton X-100, 0.5% Sodium deoxycholate) were added. Tubes were centrifuged for 20 min at 13,000 rpm at 4°C. The supernatant was then stored at -80°C for further analysis. In addition, the whole blood samples were collected using anticoagulant free tube and were centrifuged at 3,000 rpm for 10 min at 4°C. The supernatant was then stored at -80°C for further analysis. Cell culture supernates were collected and centrifuged at 1000 × g for 5 minutes at 4°C. Then, cytokines including tumor necrosis factor (TNF)-*α*, interleukin (IL)-6, IL-1*β*, and IL-10 were determined using commercial enzyme-linked immunosorbent assay (ELISA) kits (HS Quantikine; R&D Systems, Minneapolis, MN, USA) following manufacturers' guidelines.

### 2.6. Western Blot Assays

Cell proteins were extracted using RIPA buffer, and concentration was measured by the BCA protein assay kit (ab102536, Abcam, Shanghai, China). Tissue protein (50 *μ*g) or cellular protein lysates (30 *μ*g) were separated by electrophoresis in 12% PAGE gels (BeyoGel™, Beyotime, Shanghai, China) following a standard protocol. Primary antibody against CXCL14 (ab137541) was purchased from Abcam (Shanghai, China). GAPDH (ab181602, Abcam) was used as loading control.

### 2.7. Real-Time PCR Analysis

Real-time PCR was performed to measure CXCL14 mRNA expressions. Total RNA were extracted from cells and renal tissues using RNeasy RNA isolation kit (Qiagen, Valencia, CA). Real-time PCR was performed using ABI prism 7700 (Applied Biosystems, Foster City, CA) as previously described [19]. The sequences for primers were used as follows: CXCL14 forward, 5′-GGCCCAAGATCCGCTACA-3′ and reverse, 5′-TGGGTACTTTGGCTTCATTTCC-3′; inducible nitric oxide synthase (iNOS) forward 5′-GAATTCCCAGCTCATCCGGT-3′ and reverse, 5′-GGTGCCCATGTACCAACCGGT-3′; arginase-1 (Arg-1) forward, 5′-CCGCAGCATTAAGGAAAGC-3′ and reverse, 5′-CCCGTGGTCTCTCACATTG-3′; and *β*-actin forward, 5′-CAGTAACAGTCCGCCTAGAA-3′ and reverse, 5′-GATTACTGCTCTGGCTCCTA-3′.

### 2.8. Blood Bacterial Load

Blood samples were obtained from the vena cava at 12 hours after CLP surgery (*n* = 6 per group). Following 1 : 3 dilution in sterile PBS, 100 *μ*L mixture was plated on 5% blood agar plates. Plates were incubated for 24 hours at 37°C, and then colony enumeration was counted.

### 2.9. Blood Sample Analysis

Blood samples were collected by tail-vein bleeding. Serum creatinine was measured using an i-STAT 1 Analyzer (Abbott, Kyoto, Japan).

### 2.10. Measurements of Renal CXCL14 and NGAL Expressions

The measurements of renal CXCL14 expressions have been performed by ELISA. Renal cortex collection, tissue lysates preparation, and protein concentration measurements were performed as described above. As previously described [19], ELISA plates were coated with 10 *μ*g/ml monoclonal anti-FLAG Ab (Sigma-Aldrich, St. Louis, MO). Tissue lysates were diluted to 0.3 mg/ml and added into the plates. Polyclonal antimouse CXCL14 (R&D Systems, Minneapolis, MN) was used as the detecting Ab. In order to evaluate kidney injury, renal neutrophil gelatinase-associated lipocalin (NGAL) protein levels were measured using a high sensitivity enzyme-linked immunosorbent assay (ELISA) kits (R&D Systems Inc., Minneapolis, MN, USA) following manufacturer's guidelines.

### 2.11. Histological Analysis

Renal specimens were fixed in 10% formalin, embedded in paraffin, cut into 4-micrometer sections, and stained with hematoxylin-eosin. Renal damages were evaluated as previously described [8], and specimens were scored ranging from 0 (no damage) to 10 (maximal damage).

### 2.12. Survival Analysis

Animals (*n* = 10 per group) were subjected to sham, CLP, CXCL14-Tg, and CXCL14 − Tg + CLP groups as previously stated. Animals were then monitored after surgical procedure (CLP) for 72 hours. The survival rate during observation was calculated.

### 2.13. Statistical Analysis

The data were expressed as means ± SD. Statistical analysis was performed using the SPSS 17.0 software package (SPSS Inc., Chicago, USA). Comparison between groups were determined using one-way analysis of variance (ANOVA) followed by unpaired Student *t* tests. Histological scores were compared using the Steel-Dwass test followed by the Kruskal-Wallis test. Correlation analysis was performed by Pearson correlation analysis. Survival analysis was presented in Kaplan-Meier curves, and comparisons between two groups were performed by the log-rank test. Two-tailed *p* values less than 0.05 were considered significant.

## 3. Results

### 3.1. Renal CXCL14 Expression Was Decreased in the Kidney after CLP and Negatively Correlated with Serum Creatinine Levels and Cytokine Productions in Mice

**Figure 1:**
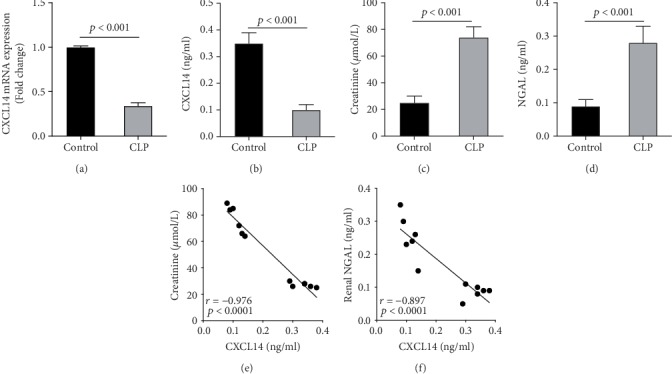
CLP downregulated renal CXCL14 expression in wild-type mice (*n* = 6 in each group). (a) RT-PCR and (b) ELISA assays suggested that CXCL14 mRNA and protein expressions were downregulated in the kidney at 12 hours after CLP; CLP induced kidney injury as assessed by the (c) increased serum creatinine levels and (d) upregulated renal NGAL expressions; correlation analysis showed that (e) serum creatinine and (f) renal NGAL were negatively associated with the expression levels of renal CXCL14. Bars display means ± SD, and measurements were repeated three times. CLP = cecal ligation and puncture; NGAL = neutrophil gelatinase-associated lipocalin.

**Figure 2:**
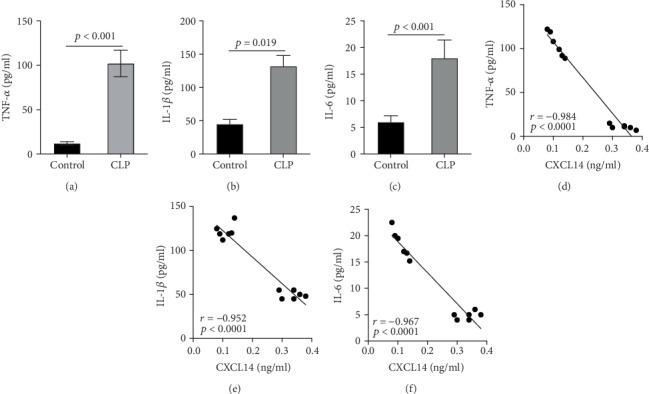
Renal cytokines were negatively associated with renal CXCL14 expression in wild-type mice (*n* = 6 in each group). Proinflammatory cytokines including (a) TNF-*α*, (b) IL-1*β*, and (c) IL-6 in the kidney were significantly increased at 12 hours after CLP; correlation analysis showed that (d) TNF-*α*, (e) IL-1*β*, and (f) IL-6 were negatively associated with renal CXCL14 expressions. Bars display mean ± SD, and measurements were repeated three times. CLP = cecal ligation and puncture.

We first confirmed the downregulation of mRNA and decreased protein of CXCL14 in the kidney of mice at 12 hours after CLP. As seen in Figures 1(a) and 1(b), renal CXCL14 expression was significantly decreased at 12 hours after CLP in WT mice (*p* < 0.05, respectively). Serum creatinine and renal NGAL expression were significantly increased after CLP (Figures 1(c) and 1(d)). In addition, the correlation analysis showed that renal CXCL14 expressions were negatively associated with the serum levels of creatinine (*r* = −0.976, *p* < 0.0001) and renal NGAL expressions (*r* = −0.897, *p* < 0.0001) (Figures 1(e) and 1(f)). As seen in Figure 2, CLP induced marked increases of renal proinflammatory cytokines including TNF-*α*, IL-6, and IL-1*β* in WT mice (Figures 2(a)-2(c)). In addition, renal CXCL14 protein expressions were negatively associated with the production of TNF-*α* (*r* = −0.984, *p* < 0.0001), IL-6 (*r* = −0.967, *p* < 0.0001) and IL-1*β* (*r* = −0.952, *p* < 0.0001) in the kidney of mice after CLP (Figures 2(d)-2(f)).

### 3.2. CXCL14 Overexpression Resulted in Decreased Bacterial Load Together with Downregulated Expression of Cytokines

**Figure 3:**
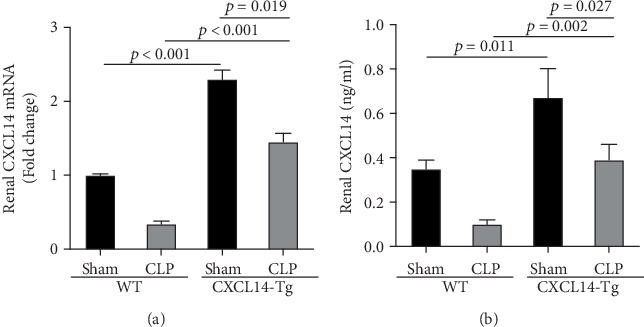
CXCL14 overexpression in the kidney (*n* = 6 in each group). (a) RT-PCR and (b) ELISA assays were performed to measure CXCL14 mRNA and protein expressions, respectively. CXCL14 expression in the kidney of CXCL14-Tg mice were significantly higher than that of wild-type (WT) mice at 12 hours after CLP. Bars display mean ± SD, and measurements were repeated three times. CLP = cecal ligation and puncture.

**Figure 4:**
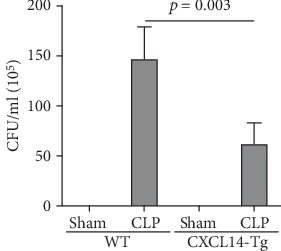
CXCL14 overexpression inhibited blood bacterial load (*n* = 6 in each group). The number of bacterial colonies was counted after 24 h of intubation. Data were expressed as means ± SD, and measurements were repeated three times. WT = wild-type; CFU = colony-forming units; CLP = cecal ligation and puncture.

**Figure 5:**
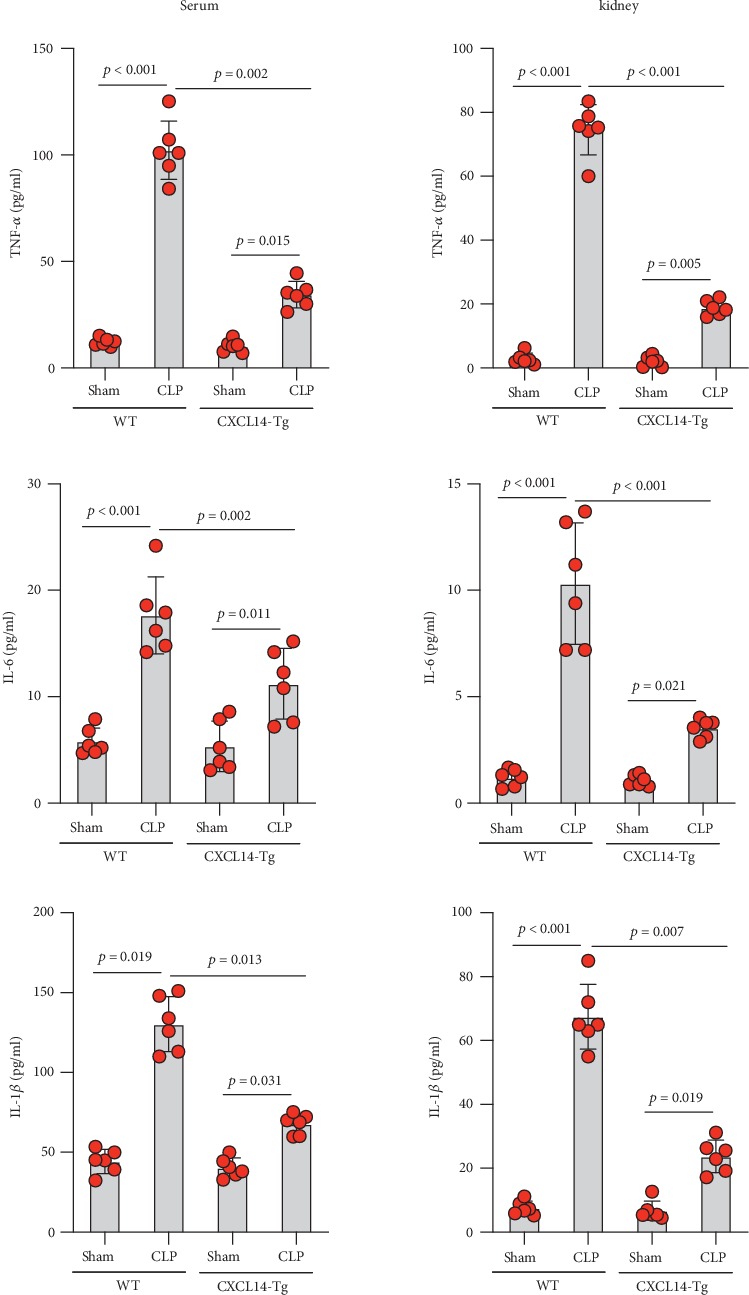
CXCL14 overexpression attenuated CLP-induced systemic and renal inflammation (*n* = 6 in each group). At 12 hours after CLP, systemic (serum) and renal proinflammatory cytokines including TNF-*α*, IL-1*β*, and IL-6 were significantly lower in CXCL14-Tg mice than that in WT mice. Bars display mean ± SD, and measurements were repeated three times. WT = wild-type; CLP = cecal ligation and puncture.

Our results demonstrated that renal CXCL14 mRNA levels (Figure 3(a)) and protein expressions (Figure 3(b)) were significantly increased in the kidney of CXCL14-Tg mice as compared with the wild-type (WT) mice, indicating an overexpression of CXCL14. At 12 hours after CLP, renal CXCL14 mRNA and protein expressions in the CXCL14 − Tg + CLP group was significantly higher than that in CLP group (*p* < 0.05, respectively). In addition, CXCL14 expressions in the CXCL14 − Tg + CLP group were significantly decreased as compared with the CXCL14-Tg group (*p* < 0.05, respectively). Blood bacterial load in the CXCL14 − Tg + CLP group was significantly decreased as compared with the CLP group (*p* < 0.05) (Figure 4). In addition, systemic and renal cytokines in the CXCL14 − Tg + CLP group were significantly decreased as compared with CLP group (*p* < 0.05, respectively) (Figure 5).

### 3.3. CXCL14 Overexpression Decreased M1 Polarization but Increased M2 Polarization *in vivo* and *in vitro*

**Figure 6:**
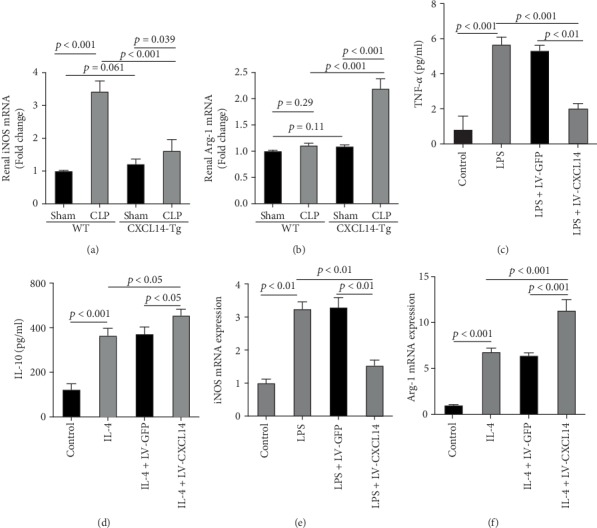
CXCL14 overexpression inhibited M1 polarization and improved M2 polarization *in vivo* and *in vitro*. (a) Renal iNOS and (b) Arg-1 mRNA expressions were determined by RT-PCR. RAW264.7 received lentivirus infections for 48 hours and were then treated with *lipopolysaccharides* (LPS, 100 ng/ml, 8 h) or IL-4 (10 ng/ml, 12 h). Then, (c) TNF-*α* and (d) IL-10 expressions were measured by ELISA kits. (e) iNOS and (f) Arg-1 mRNA expressions were determined by RT-PCR.

Since animal models demonstrated that the macrophage is a major contributor to the inflammatory response to AKI, we next determined the effects of CXCL14 on macrophage polarization *in vivo* and *in vitro*. As seen in Figures 6(a) and 6(b), RT-PCR showed that renal iNOS, a major M1 macrophage marker, was significantly decreased in the CXCL14 − Tg + CLP group as compared with the CLP group (*p* < 0.001, respectively). In addition, the expression of M2 marker Arg-1 in injured kidneys was significantly increased in the CXCL14 − Tg + CLP group even in the early stage of sepsis-associated AKI as compared with the CLP group (*p* < 0.001, respectively). Moreover, as compared with the sham group, iNOS expressions were significantly increased in the CLP group (*p* < 0.05). However, no significant differences were observed in Arg-1 expressions between the sham and CLP group (*p* = 0.029). We also determined the effects of CXCL14 overexpression on macrophage polarization *in vitro*. Our results showed that CXCL14 overexpression inhibited M1 polarization but increased M2 polarization as reflected by decreased expressions of TNF-*α* and iNOS and increased expressions of IL-10 and Arg-1, respectively (*p* < 0.05, respectively) (Figures 6(c)-6(f)). Together, these results suggested that CXCL14 overexpression decreased M1 polarization but increased M2 polarization *in vivo* and *in vitro*.

### 3.4. CXCL14 Overexpression Attenuated CLP-Induced AKI and Improved Animal Short-Term Survival Rate

**Figure 7:**
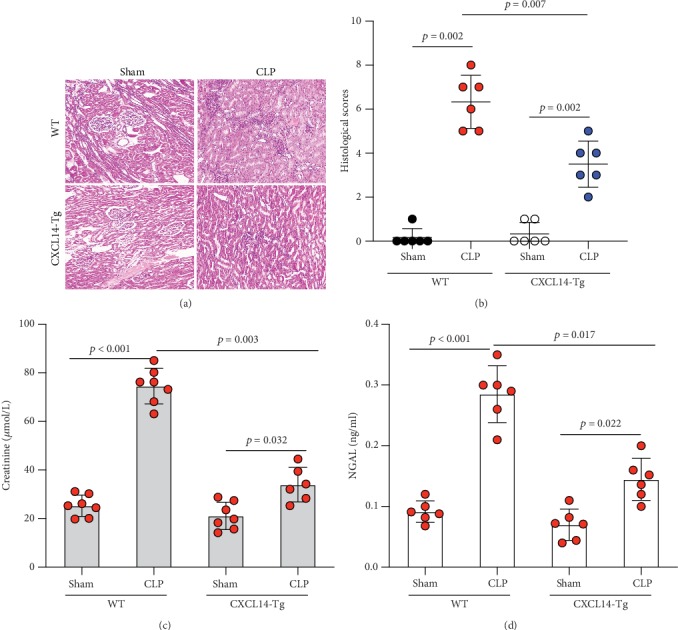
CXCL14 overexpression attenuated CLP-induced AKI in mice (*n* = 6 in each group). (a) Histological analysis after H&E stain and (b) histological scores suggested that CXCL14 overexpression diminished CLP-induced AKI with significantly lower histological scores. In addition, CXCL14 overexpression decreased (c) serum creatinine levels and reduced (d) renal NGAL expression at 12 hours after CLP. Bars display means ± SD, and measurements were repeated three times. CLP = cecal ligation and puncture; AKI = acute kidney injury; NGAL = neutrophil gelatinase-associated lipocalin.

**Figure 8:**
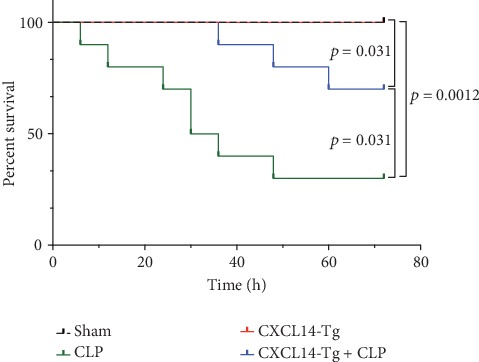
Survival analysis. Ten animals in each group were enrolled for a 72-hour survival analysis. CLP = cecal ligation and puncture.

CXCL14 overexpression improved renal histology (Figures 7(a) and 7(b)) and attenuated CLP-induced AKI as evaluated by serum creatinine levels (Figure 7(c)) and renal NGAL expressions (Figure 7(d)). In detail, the histological scores in the CXCL14-Tg mice were significantly lower than that in WT mice at 12 hours after CLP (*p* = 0.007). In addition, serum creatinine levels and renal NGAL expressions after CLP were significantly downregulated by CXCL14 overexpression (*p* < 0.05, respectively). Moreover, survival analysis demonstrated a significantly lower mortality in the CXCL14 − Tg + CLP group than that in the CLP group (70% vs. 30%, *p* = 0.031) (Figure 8).

## 4. Discussion

The major findings of this study can be summarized as follows: (1) systemic and intrarenal proinflammatory cytokines were upregulated together with decreased renal CXCL14 expression in mice; (2) renal CXCL14 expression was negatively associated with the production of cytokines and the severity of AKI in septic mice; (3) overexpression of CXCL14 attenuated sepsis-associated AKI together with improved survival outcome; and (4) CXCL14 overexpression decreased M1 polarization but increased M2 polarization both *in vivo* and *in vitro*.

The release of cytokines and the recruitment of neutrophils to the site of injury are considered as the hallmark of inflammation, which is an important biologic response that is essential for eliminating pathogens and repairing tissue after injury; however, excessive inflammation induces autoimmune disorders and promotes tissue damage [21]. This concept is especially notable in severe sepsis, where the excessive production of cytokines causes microcirculation dysfunction, tissue injury, and multiple organ failure [22]. Specifically, numerous evidences suggest that AKI associates intrarenal and systemic inflammation [23]. In the present study, CLP resulted in an excessive production of systemic and intrarenal proinflammatory cytokines together with markedly increased serum creatinine levels and renal NGAL expressions, which were in line with previous findings. Importantly, CXCL14 overexpression attenuated CLP-induced AKI and improved survival outcome of septic animals.

In the past decade, a growing body of evidence suggested that sepsis-induced immune response participates in the activation of both proinflammatory and anti-inflammatory mechanisms [24, 25]. CXCL14 has been reported to be involved in antimicrobial immunity prior to the establishment of inflammation [18]. In addition, CXCL14 is expressed either constitutively or following stimulus in both immune and nonimmune cells. For example, freshly isolated human peripheral blood mononuclear cells do not exhibit CXCL14 expression, whereas CXCL14 mRNA is detected in monocytes after LPS treatments [12]. However, some other studies have reported that CXCL14 expression could be inhibited by TNF-*α* and LPS [16, 17]. In the present study, we observed that renal CXCL14 expression was decreased together with excessive production of cytokines including TNF-*α*, IL-1*β*, and IL-6 in septic mice. Correlation analysis suggested a negative association between all these proinflammatory cytokines and renal CXCL14 expression. A very recent study reported that CXCL14 analogs including CXCL14-C17-a2 and CXCL14-C17-a3 effectively inhibited the production of TNF-*α* and IL-6 from LPS-stimulated RAW264.7 cells [26]. In addition, it has been widely accepted that macrophage is one of the major sources of inflammatory cytokine production during sepsis [27], and sepsis-associated AKI is related to the polarization of proinflammatory M1 or anti-inflammatory M2 [28]. M1 macrophage contributes to the excessive production of proinflammatory cytokines and AKI, but M2 macrophage accelerates tissue repair and inhibits proinflammatory cytokine production [29]. Currently, iNOS has been used as a marker of M1 macrophages [30], and the expression of Arg1 has been considered as a classical inducer of M2 gene expression [31]. In the present study, we found that CXCL14 overexpression decreased M1 macrophage polarization but increased M2 polarization, resulting in decreased proinflammatory cytokine (TNF-*α*) production and increased anti-inflammatory cytokine (IL-10) production. In addition, we observed that CXCL14 overexpression also decreased bacterial load in septic mice, which could be also related to M1/M2 polarization [32]. Therefore, it is reasonable to speculate that CXCL14 overexpression attenuate renal inflammation probably through the modulation of M1/M2 polarization.

There are three major limitations in this study. First, correlation analysis suggested a negative association between proinflammatory cytokines and renal CXCL14 expression. Cell study showed that CXCL14 overexpression inhibited cytokine production in LPS-stimulated RAW264.7 cells. However, *in vitro* experiments should also be performed to identify whether cytokines are able to downregulate CXCL14 expressions. Second, the effects of CXCL14 overexpression on cytokine production have been investigated in this study. However, the impacts of CXCL14 knockout/knockdown on proinflammatory cytokine expressions have not been performed. Finally, although we observed that CXCL14 overexpression inhibits proinflammatory cytokine production and modulates M1/M2 polarization in RAW264.7 cells, primary macrophages have not been isolated to identify its capacity in M1/M2 differentiation.

## 5. Conclusion

Collectively, our results suggested that the loss of renal CXCL14 expression probably contributed to the excessive proinflammatory cytokine production in the kidney. In addition, the overexpression of CXCL14 increased M2 polarization and attenuated intrarenal inflammation, which resulted in improved renal function and survival outcome of septic mice. Together, these findings first demonstrated CXCL14 overexpression attenuate sepsis-associated AKI probably through the modulation of M1/M2 polarization.

## Data Availability

The data are available from the corresponding author upon reasonable request.
